# Oral Inoculation of Specific-Pathogen-Free Chickens with Chicken Anemia Virus Induces Dose-Dependent Viremia and Transient Anemia

**DOI:** 10.3390/pathogens8030141

**Published:** 2019-09-07

**Authors:** Suttitas Tongkamsai, Meng-Shiou Lee, Yi-Lun Tsai, Hsyang-Hsun Chung, Guan-Hua Lai, Jai-Hong Cheng, Ming-Chu Cheng, Yi-Yang Lien

**Affiliations:** 1 Department of Veterinary Medicine, College of Veterinary Medicine, National Pingtung University of Science and Technology, Pingtung 91201, Taiwan (S.T.) (Y.-L.T.) (H.-H.C.); 2 Faculty of Veterinary Medicine, Rajamangala University of Technology Tawan-ok, Chonburi 20110, Thailand; 3 School of Chinese Pharmaceutical Sciences and Chinese Medicine Resources, China Medical University, Taichung 40402, Taiwan (M.-S.L.) (G.-H.L.); 4 Research Center of Animal Biologics, National Pingtung University of Science and Technology, Pingtung 91201, Taiwan; 5 Center for Shockwave Medicine and Tissue Engineering, Department of Medical Research, Kaohsiung Chang Gung Memorial Hospital and Chang Gung University College of Medicine, Kaohsiung 833, Taiwan

**Keywords:** chicken anemia virus, viremia, anemia

## Abstract

Chicken infectious anemia caused by chicken anemia virus (CAV) is a very important immunosuppressive disease in chickens. The horizontal spread of CAV in field chickens has been confirmed mainly through oral infection in our published article. Anemia is the main symptom of this disease. Studies by other scientists have shown that infection of CAV in 1-day-old chicks can cause anemia, and the degree of anemia is directly proportional to the dose of infectious virus. However, the pathogenesis of oral inoculation of CAV in older chickens is still not well understood. The purpose of this study was to determine whether 3-weeks-old specific-pathogen-free (SPF) chickens infected with different viral doses in oral route would cause anemia, as well as other signs associated with age-resistance. The experimental design was divided into a high-dose inoculated group (10^6^ 10_50_), low-dose inoculated group (10^3^ TCID_50_), and non-virus inoculated control group, and 12 birds in each group at the beginning of the trial. The packed cell volumes (PCVs), CAV genome copies in tissues, CAV titer in peripheral blood fractions, and serology were evaluated at 7, 14, and 21 days post-infection (dpi). Virus replication and spread were estimated using quantitative polymerase chain reaction (qPCR) and viral titration in cell culture, respectively. The results showed that the average PCVs value of the high-dose inoculated group was significantly lower than that of the control group at 14 dpi (*p* < 0.05), and 44.4% (4/9) of the chickens reached the anemia level (PCVs < 27%). At 21 dpi, the average PCV value rebounded but remained lower than the control group without significant differences. In the low-dose inoculated group, all birds did not reach anemia during the entire trial period. Peripheral blood analysis showed that the virus titer in all erythrocyte, granulocyte and mononuclear cell reached the peak at 14 dpi regardless of the high-dose or low-dose inoculated group, and the highest virus titer appeared in the high-dose inoculated group of mononuclear cell. In the low-dose inoculated group, CAV was detected only at 14 dpi in erythrocyte. Taken together, our results indicate that the older birds require a higher dose of infectious CAV to cause anemia after about 14 days of infection, which is related to apoptosis caused by viral infection of erythrocytes. In both inoculated groups, the viral genome copies did not increase in the bone marrow, which indicated that minimal cell susceptibility to CAV was found in older chickens. In the low-dose inoculated group, only mononuclear cells can still be detected with CAV at 21 dpi in seropositive chickens, indicating that the mononuclear cell is the target cell for persistent infection. Therefore, complete elimination of the CAV may still require the aid of a cell-mediated immune response (CMI), although it has previously been reported to be inhibited by CAV infection. Prevention of early exposure to CAV could be possible by improved hygiene procedures.

## 1. Introduction

Chicken anemia virus (CAV) belongs to the genus Gyrovirus of the family Anelloviridae [1]. The virus induces chicken infectious anemia (CIA), which is characterized by severe anemia in young chicks and immunosuppression in all chickens. CAV targets hemocytoblasts in the bone marrow and T-lymphocyte precursors in the thymus [2]. It is commonly believed that infection in chickens older than three weeks of age occurs in the absence of clinical signs due to the rapid development of antibodies [3]. Wani et al. [4] studied the hematological parameters of 6-week-old chickens with intramuscular CAV inoculation. They found that the mean packed cell volume of the CAV-inoculated group decreased, and the chickens were considered anemic after 14 days post-infection. The pathogenesis of CAV was shown to depend on the challenge dose. McNulty et al. [5] demonstrated that the minimum dose of CAV that caused anemia in a high proportion of 1-day-old inoculated chickens was above 10^3^^.3^ 50% tissue culture infectious dose (TCID_50_). In Taiwan, most field CAV cases were detected in older chickens with transient poor performance [6]. Dren et al. [7] examined the pathogenesis of CAV infection in 6-week-old chickens with different inoculation doses. They demonstrated that in the high-dose CAV-inoculated group viremia and antibodies were detected earlier than they were in the low-dose group. These results suggest that CAV replication in older chickens probably depends on the exposure dose. Natural exposure to CAV in older chickens is generally by the fecal-oral route. Additionally, anemia was found in 3-week-old chickens after oral inoculation with CAV [8,9]. Blood cells were reported to have the highest detected CAV load compared with the thymus and the bone marrow [10], and the virus could be isolated from these cells throughout the experimental period [11]. These results indicated that blood cells are one of the sources of CAV infection. However, there are limited data on which type of blood cell mostly carries CAV infection. The pathogenesis of oral inoculation of CAV in older chickens is still not well understood. This study aimed to describe the association between measurable clinical parameters and viral replication in 3-week-old chickens inoculated orally with different doses of CAV. For this purpose, the packed cell volumes (PCVs), viral load, viremia, and antibody titer were evaluated.

## 2. Results

### 2.1. Hematological Changes

**Figure 1:**
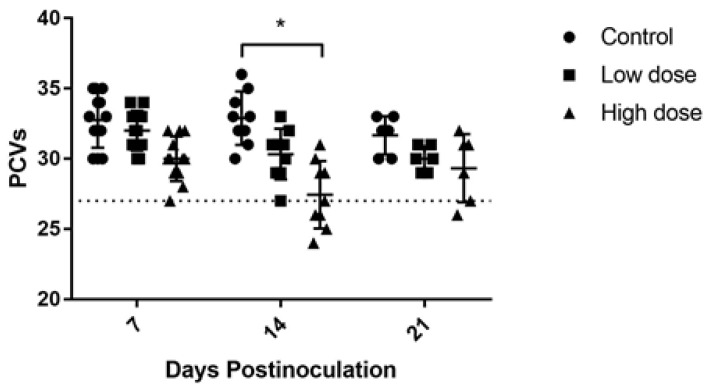
The effect of chicken anemia virus (CAV) inoculation on packed cell volumes (PCVs) in groups with different inoculum doses. The dotted line represents the boundary of anemia (PCVs < 27%). The dots represent each PCV of chickens; * *p* < 0.05 indicates a significance in the percentage of anemic chickens between groups.

To observe the incidence of anemia due to CAV infection in inoculated chickens, the PCVs were determined. Throughout the experimental period, nonsignificant differences were observed within the control and the low-dose inoculated group. The packed cell volumes were significantly lower (*p* < 0.05) at 14 days post inoculation (dpi) while compared with those at 7 dpi in the high-dose inoculated group. At 7 dpi, compared with the uninoculated control group, the high-dose inoculated group showed a significantly low PCVs (*p* < 0.05), while the low-dose inoculated group had no significant difference. At 14 dpi, the PCVs in both inoculated groups were significantly lower (*p* < 0.05) compared with the control group. There were no significant differences among the three groups at 21 dpi. By the standard of chicken anemia (PCVs < 27%), anemic chickens were absent in the control group and in both inoculated groups at 7 dpi. At 14 dpi, a significantly high percentage of anemic chickens (4/9, 44.4%) were detected in the high-dose inoculated group compared with the low-dose inoculated group and control group. One anemic chicken was found in the high-dose inoculated group at 21 dpi but showed no significant difference with the other two groups (Figure 1).

### 2.2. Standardization of qPCR for Viral Load Detection

**Figure 2:**
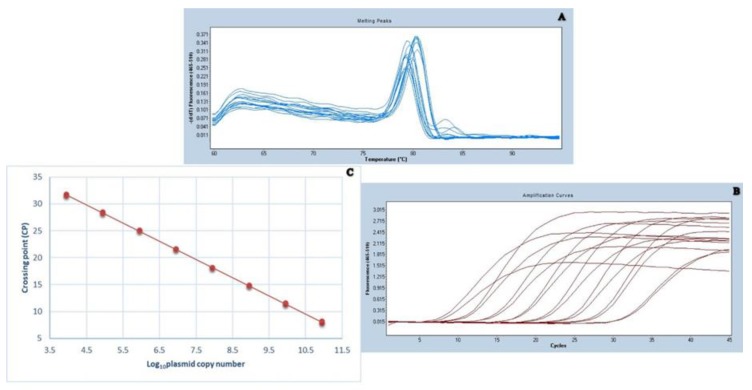
Dissociation curve of the amplified PCR product (65 bp size) in melting curve analysis (**A**). Amplification plots of 10-fold serially diluted (10^−8^ to 10^−1^) *ORF*2-pGEM-T Easy linearized recombinant plasmid in duplicate (**B**). Standard curve generated from 10-fold serial dilutions as described in Figure 2B (**C**).

The standard curve was generated from a constant linear correlation between the amount of 10-fold dilutions of *ORF2*-pGEM-T Easy plasmid and crossing point (CP) values. According to the plotted standard curves, the linear equation was y = −3.398x + 10.70. The R^2^ index value, which represents the correlation coefficient of a standard curve in a linear regression analysis, was 0.9927. The efficiency of the amplification was 1.969. The standard curves are shown in Figure 2.

### 2.3. CAV Genome Quantification

**Figure 3:**
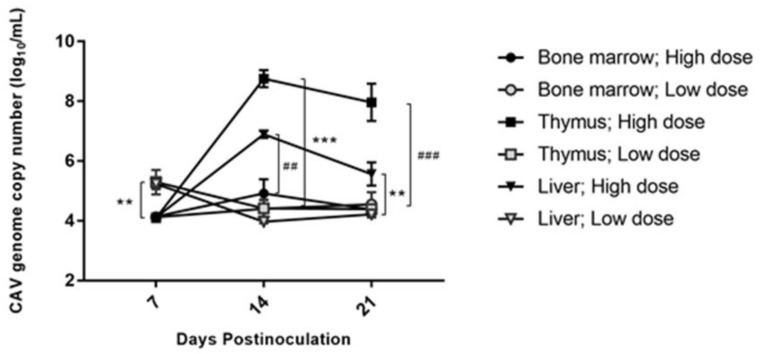
Viral load in different organs of both inoculated groups. Values are represented as the mean ± SD of CAV genome copy number (log_10_/mL) within tissue mixture; ** *p* < 0.01 and *** *p* < 0.001 indicate significant differences with the same organ between the groups. ^##^ *p* < 0.01 and ^###^ *p* < 0.001 indicate significant differences compared with the different organs in the same group.

The CAV genome was quantified in sacrificed infected chickens at 7-day intervals up to 21 dpi. The bone marrow was shown to have a low viral load compared with thymus and liver in both inoculated groups throughout the experiment period. At 7 dpi, the thymus of the low-dose inoculated group was found to have a significantly high mean viral load (*p* < 0.01) compared with that in the thymus of the high-dose inoculated group. At 14 dpi, in the high-dose inoculated group, the highest mean viral load was detected in the thymus (log_10_ 8.75 ± 0.28) and the peak viral load was observed at this time point in all three organs tested. The viral load in the thymus of the high-dose inoculated group was remarkably higher (*p* < 0.001) than that in the thymus of the low-dose inoculated group. The liver of the high-dose inoculated group was found to have highly significantly (*p* < 0.01) more CAV genome copy numbers than those of the bone marrow in the same group. At 21 dpi, the trend of a drop in the mean viral load was observed. In the bone marrow of the high-dose inoculated group, the mean viral load was still significantly lower (*p* < 0.001) compared with that in the thymus of the same group. The CAV genome copy number in the liver in the high-dose inoculated group remained higher than that in the liver of the low-dose inoculated group (Figure 3)

### 2.4. Virus Titration in Blood

The virus titer in blood was recovered by virus re-isolation into MSB-1 cells. After, whole blood was separated into four fractions, including plasma, mononuclear cells, granulocytes, and erythrocytes. In the high-dose inoculated group, CAV could be detected by either virus re-isolation or PCR except for the plasma on 14 and 21 dpi. The erythrocytes of the high-dose inoculated group at 14 dpi were found to be significantly increased (*p* < 0.05) from 7 dpi. The virus titer did not change significantly in the mononuclear cells throughout the experimental period. In the low-dose inoculated group, CAV was not found in plasma throughout the experimental period, while the virus could be detected at different dpi in mononuclear cells, granulocytes and erythrocytes by virus re-isolation or PCR. CAV titer in mononuclear cells was not significantly different at 14 dpi compared with 21 dpi. CAV titer was not detected in the uninoculated control group. At 14 dpi, the mononuclear cells of the high-dose inoculated group had the highest viremia. At this time point, the mononuclear cells of the high-dose inoculated group was found to have a significantly high (*p* < 0.05) CAV titer compared with the granulocytes of the same group, and were also highly significantly different (*p* < 0.01) compared with the granulocytes of the low-dose inoculated group (Table 1).

### 2.5. Humoral Immune Response

**Figure 4:**
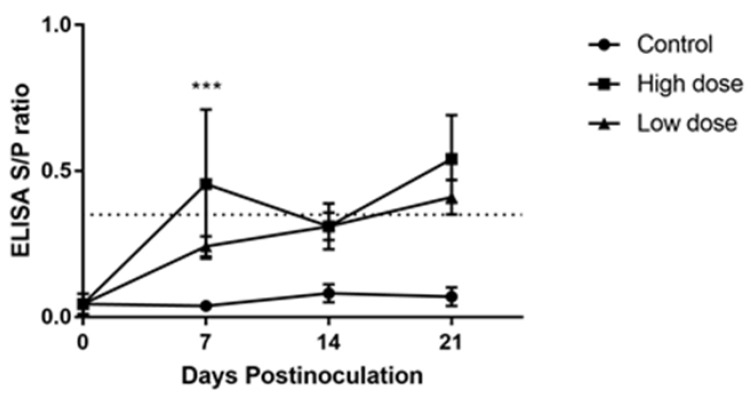
The effect of CAV inoculation on specific antibody levels in groups with different inoculum doses. The dotted line represents the cutoff titer between negative and positive antibody status. The values are represented as the means ± SD; *** *p* < 0.001 indicates a significant difference in the high-dose inoculated group compared with the low-dose inoculated group.

The influence of CAV on the induction of antibodies in 3-week-old chickens was monitored using ELISA. All chosen chickens had no detectable CAV antibodies at the time of inoculation. The antibody titer in both inoculated groups was significantly different compared with the control group throughout the experimental period. At 7 dpi, the mean CAV-specific antibody levels in the high-dose inoculated group was significantly higher (*p* < 0.001) than that in the low-dose inoculated group. At 14 and 21 dpi, there was no difference of antibody titer between the inoculated groups. In the high-dose inoculated group, a significant decrease (*p* < 0.05) in the antibody titer was observed from 7 to 14 dpi and then increased toward 21 dpi. The antibody titer was increased from 7 to 21 dpi in the low-dose inoculated group, and the mean of antibody levels at 21 dpi was significantly higher (*p* < 0.05) than that at 7 dpi. Based on the antibody status, the low-dose inoculated group remained negative for CAV, while the high-dose inoculated group was positive at 7 dpi. The antibody titer of all inoculated chickens reached its maximum level at 21 dpi. The chickens of the uninoculated control group maintained seronegative status for CAV-specific antibodies until the end of the experiment (Figure 4).

## 3. Discussion

In the present work, we report quantitative data that represent CIA as seen in the field, in which the amount of virus shedding is difficult to predict. The relationship between the viral load in various organs and the viremia of orally infected 3-week-old chickens were established. Moreover, the antibody titer following different inoculation doses was described. 

Our experimental results obtained at 14 days after high-dose CAV inoculation showed that some chickens developed anemia. These observations are in agreement with those reported by Wani et al. [4], who have shown that inoculation with CAV in older chickens is able to induces mild anemia. The transient presence of anemia does not support the age-related resistance hypothesis. The virus dose has been reported to influence the severity of anemia in younger chickens [5]. The virus causes suppression of hematopoietic precursor cell proliferation in the bone marrow. Nevertheless, oral CAV inoculation of mature chickens substantiated that CAV antigens are rarely found in the bone marrow, even in anemic chickens [8]. The present findings confirmed this observation with data showing that viral load in the bone marrow of both inoculated groups at 14 dpi was low throughout the experiment, and the high-dose inoculated group was not significantly different from the low-dose inoculated group, in which anemic chickens were absent. This phenomenon indicates that the hemocytoblasts in the bone marrow are less susceptible to CAV infection in older chickens and that immature myeloid cells might not be the only cells sensitive to the virus infection in the erythrocyte lineage. Replication of CAV in the erythrocytes was detected at 14 dpi in the high-dose inoculated group, and it significantly increased from 7 dpi. This result is interpreted to mean that mature erythrocytes may be infected with CAV, and then apoptosis of these cells may lead to anemia. A similar result that blood cells are sensitive to CAV infection was previously reported [11]. One conclusion is that anemia may also appear in older chickens with CAV inoculation. There is a positive correlation between the virus inoculum dose and the virus titer in the erythrocytes, which means that the potential of the virus to reduce the number of erythrocytes in older chickens is dose dependent. 

The early target for CAV replication is hemocytoblasts in the bone marrow and thymocytes in the thymic cortex [12]. The present study showed that the viral load of the thymus in the high-dose inoculated group was significantly lower than that in the low-dose inoculated group at 7 dpi. This result demonstrates that virus infection was associated with CAV specific antibody production. At this time point, the antibody titer of the high-dose inoculated group was positive. It has been suggested that a high-dose CAV inoculation may induce a high level of antibody titer and that titers above the positive cutoff level are generally considered protective [13]. Unexpectedly, the viral load in the liver in the high-dose inoculated group increased with the same trend as in the thymus at 14 dpi and was significantly different from the viral titer in the bone marrow of the same group. There are two possible reasons: first, the organ was not perfused before harvest, and some of the virus observed in the liver may have been circulating in the blood [14]. Secondly, Markowski-Grimsrud and Schat [15] suggested that CAV possibly infects secondary lymphoid organs. As far as we know, the liver is a granulopoietic organ in chickens [16]. This observation was justified by the results in Table 1, in which the virus distribution demonstrated that CAV could be detected in the granulocyte fraction of both inoculated groups and the titer increased at 14 dpi. The CAV load in the thymus of both inoculated groups reached the maximum level at 14 dpi and then decreased at 21 dpi, corresponding with the humoral immune response. At 21 dpi in the high-dose inoculated group, the viral load in the thymus remained high, coincident with the notable CAV titer in the mononuclear fraction at the same time point. These studies indicate that the thymus is a source of CAV-carrying cells and that infected mononuclear cells spread throughout the body via blood circulation [17]. The liver might be a source of infected granulocyte dissemination. Further experiments are required to elucidate the validity of this assumption. 

The present study examined the virus titer in blood components and found that CAV could be recovered from the plasma in the high-dose inoculated group at 7 dpi. It has been described that many susceptible cells are infected by CAV and then release the viable virus by apoptosis. These findings were supported by Kuscu and Gürel [18] showing that CAV replication is first detectable in infected cells at 4 dpi. A previous study showed that CAV could not be detected in the plasma of 40-day-old inoculated chickens [11]. This discrepancy may be due to the difference in the age of the infected chickens—younger chickens were inoculated in our experiment than in the previous work. Consistent with the observation that lymphocytes and monocytes are the primary target cells, the virus titer in the mononuclear fraction of both inoculated groups was increased at 14 dpi and was identical to the seronegative status. It has been reported that CAV is able to persist in mononuclear cells derived from multiple lymphoid organs [19]. Imai et al. [11] suggested that virus infectivity was related to blood cell fractions. At 21 dpi, CAV infectivity was still detected in all of the cell fractions in the high-dose inoculated group, while only the mononuclear cells carried the virus in the low-dose inoculated group. This result illustrated that the transmission of CAV-infected cells likely depends on the inoculation dose. 

Based on the resistance to CAV infection of B lymphocytes, the control of CAV replication requires a humoral immune response [20]. At 7 dpi, the high-dose inoculation of CAV induced CAV-specific antibodies higher than in the low-dose inoculated group. Our finding on anti-CAV antibody levels supports the conclusion that the high-dose CAV inoculation of older chickens induces viral neutralizing antibodies earlier at 4 dpi [7]. In addition, we found that the presence of viral-specific antibodies was observed at the same time as the presence of viremia. Wani et al. [14] showed that high viral concentration in the blood was found even before the antibodies appeared, and the authors discussed that the primary antibody response seems to have a limited ability to clear the virus from the infected peripheral cells. Between 14 and 21 dpi, antibodies were found to be responsible for CAV elimination as a marked reduction in viremia and viral load was observed. However, the viral load in the thymus and the virus titer in the mononuclear fraction remained even after seroconversion. It has been suggested that the complete eradication of CAV requires a cell-mediated immune response, but persistent CAV infection results in immunosuppression generally found in the regression of the CD4+ T cell population [21], and a reduction in the stimulation of antigen-specific cytotoxic T-lymphocytes (CTLs) was reported in subclinical infection of CAV [15]. 

In conclusion, the present report is the first to determine a relationship between viral load in tissues and viremia in the peripheral blood. The observations in this study are consistent with those of Smyth et al. [8], who confirm that the cells of the thymus of older chickens remain susceptible to CAV infection. Moreover, we detected the virus titer in different blood components and indicated that the presence of anemia in older chickens is due to a temporary reduction in CAV-infected erythrocyte levels. Although previous reports showed that mononuclear cells are the main target cell for CAV [11,21], we further discovered that granulocytes are also susceptible to CAV infection. As for the specific cell type for CAV latency and how the virus escapes from the adaptive immune response, these require more studies to verify. Theoretically, commercial disinfectants are not effective against CAV due to high resistance. Strict hygiene was found to reduce the seroconversion rate [22]. Vaccination management and using the recommended concentration of disinfectant should be considered to minimize virus contamination in the field conditions.

## 4. Materials and Methods

### 4.1. Birds and Viral Strain

Day-old specific-pathogen-free (SPF) chicks were housed in a filtered-air, positive-pressure room. The chicks were maintained under strict isolated conditions with food and water ad libitum throughout the experimental period. High biosecurity measures were taken to avoid cross-infection. All experimental chicken studies were conducted according to an animal use protocol approved by the institutional animal care and use committees (IACUCs). Twelve chickens proved to be free of prior infection by the absence of antibody at the time of inoculation. CAV 1705PT (GenBank Accession No. MK386570) was isolated from 2-week-old native chickens in 2017 with pale combs and wattles and signs of weakness. The virus was adapted in MDCC-MSB1 cells following a generally accepted method [23]. Titration of the CAV isolate was performed; the TCID_50_ was calculated as previously reported [24].

### 4.2. Experimental Designs

Three-week-old chickens (*n* = 36) were divided into three groups—each group was comprised of 12 chickens. Groups 1 and 2 were inoculated orally with 10^6^ (high-dose) and 10^3^ (low-dose) TCID_50_ of the CAV 1705PT isolate, respectively. Infection by the oral route was shown to be capable of inducing subclinical disease in previous studies in our laboratory. Group 3 served as the uninfected control group and received mock inoculation with RPMI-1640 medium. Blood samples were collected from the remaining birds in each group from 0 to 21 dpi at 7-day intervals. Three chickens from each group were sacrificed for tissue dissection. The thymus, femur, and liver were collected and stored at −70 °C to assess the viral load.

### 4.3. Hematology

Heparinized blood samples were collected from the experimental chickens at 7, 14, and 21 dpi with CAV. The PCVs were evaluated by using the microhematocrit capillary tube method. Chickens were considered anemic if their PCV values were <27%.

### 4.4. Calculation of a Standard Curve

*ORF2* (VP2) gene of CAV was amplified using the primer sets VP2-F (5’-CGGTCCGGATCCATGCACGGAAACGGCGGACAAC-3’) and VP2-R (5’- GGTTTGGAATTCTCACACTATACGTACCGGGGC-3’). The virus-specific PCR product was ligated into the pGEM-T Easy vector (Promega, Madison, WI, USA) according to the manufacturer’s recommendations. The inserted product was transformed into competent *Escherichia* *coli* (JM109) cells. Finally, the recombinant *ORF2*-pGEM-T Easy plasmid was linearized by digestion with RsaI. The concentration of the digested DNA was calculated by measuring the absorbance at 260 nm (A_260_). A serial 10-fold dilution of the purified plasmid solution containing between 894 and 894 × 10^8^ copies was used to generate a standard curve for quantitative PCR (qPCR).

### 4.5. Tissue DNA Isolation and Viral Load Quantification

The liver, thymus, and bone marrow tissues of each experimental chicken were homogenized in phosphate-buffered saline (10% *w/v*). Isolation of viral DNA was performed using the DNeasy® blood and tissue kit (Qiagen, Hilden, Germany) following the manufacturer’s instructions. For quantification of viral load in the thymus, liver, and bone marrow samples, isolated DNA samples were subjected to real-time PCR analysis using LightCycler^®^ 480 (Roche, Basel, Switzerland). Duplicate sets of each reaction sample were prepared using 10 µL of Quantinova™ SYBR Green PCR Master Mix (Qiagen, Hilden, Germany) 0.3 µM each specific forward (QF1; 5’GAATGTGCCGGACTTGAGGA-3’) and reverse primers (QR1; 5’GGGTCGCAGGATCGCTT-3’), 5 µL of 1:10 diluted DNA samples or diluted plasmid standard and nuclease-free water to make a final reaction volume of 20 µL. Amplification conditions comprised an initial cycle at 50 °C for 2 min and at 95 °C for 2 min, followed by 45 cycles of 15 sec at 95 °C and 30 sec at 60 °C. The data were obtained as the mean ± SD of CP values. The values of the standard ranged from 0.02 to 0.68 for different dilutions. The obtained concentration values obtained were extrapolated to CAV copy numbers by using a previously described formula [25].

### 4.6. Preparation of Blood Samples and Virus Titration

Blood samples were obtained, and the plasma and blood cell fractions were separated by low-speed centrifugation. The remaining blood cells were diluted with an equal volume of phosphate-buffered saline (PBS), layered on a Lymphoprep^TM^ (Stemcell Technologies Inc., Vancouver, BC, Canada) gradient, and centrifuged at 1000× *g* for 20 min [21]. Mononuclear fractions were isolated from the interface, granulocyte fractions were collected carefully from the fraction on top of erythrocytes, and erythrocyte fractions were collected from the bottom. The granulocyte purification steps included removal of the Lymphoprep layer, the addition of ammonium chloride (Gibco Laboratories, New York, NY, USA) to the erythrocytes/granulocytes and lysis of the erythrocytes [26]. All suspensions were washed twice with a large volume of PBS. The cell pellet was resuspended in RPMI-160 medium containing antibiotic. Total cells were counted using the Natt and Herrick method [27], adjusted to 10^7^ cells per 1 mL, freeze-thawed three times and stored at −20 °C for virus assay. Flat-bottom 96-well microtiter plates were seeded with an MDCC-MSB1 cell suspension (2.5 × 10^5^ cells per plate). Ten-fold dilutions (10^0^ to 10^−5^) of infected samples were prepared, and 20 µL of each dilution was added to four replicate wells. Separated cells from uninoculated chickens served as controls. Inoculated MSB1 cells were subcultured every 2–3 days, and CAV replication in the cells was evaluated by the presence of cytopathic effect (CPE) and by quantitative PCR at the endpoint. The Reed and Muench method was used to calculate 50% endpoint log titers (log_10_TCID_50_/mL) [28].

### 4.7. Serology

Serum samples were collected from both the control and inoculated groups and were assayed for antibodies to CAV by enzyme-linked immunosorbent assay (ELISA) tests (Synbiotic Corporation, Kansas City, MO, USA) according to the manufacturer’s protocol. Serum samples with S/P ratios equal to less than 0.35 were considered negative, while S/P ratios equal to or more than 0.350 were deemed positive.

### 4.8. Statistical Analysis

Statistical analyses were carried out by using the Statistical Analysis System (SAS) version 9.4. The number of anemic chickens was compared using chi-square tests. CAV genome copies, CAV titer, and antibody titer were statistically compared by analysis of variance. *p*-values less than 0.05 were considered significant.

## Figures and Tables

**Table 1:** The effect of CAV inoculation on the virus titer in blood components (log_10_TCID_50_/mL).

Group	Blood Component	Days Post-Inoculation		
		7	14	21
High-dose inoculated	**Plasma** *	+	−	−
	Mononuclear cells	2.83 ± 1.52 ^a A^	4.50 ± 0.00 ^a B^	3.16 ± 1.52 ^a D^
	Granulocytes	+	2.00 ± 0.50 ^b C^	1.66 ± 0.28 ^b D^
	Erythrocytes	1.16 ± 0.28 ^c A^	3.50 ± 1.00 ^d B C^	2.16 ± 0.57 ^c d D^
Low-dose inoculated	**Plasma** *	−	-	-
	Mononuclear cells	+	2.00 ± 0.50 ^f C^	1.33 ± 0.28 ^f D^
	Granulocytes	+	1.50 ± 0.00 ^C^	−
	Erythrocytes	−	2.16 ± 0.76 ^C^	−

The values are represented as the mean ± SD. ^A–D^ Different superscripts within rows indicate statistically significant differences (*p* < 0.05). ^a–f^ Different superscripts within columns show statistically significant differences (*p* < 0.05). * titers adjusted per mL; ^+^ virus present and detected after the eighth passage by PCR; ^−^ virus absent after the eighth passage.
